# Obstetric and neonatal outcomes of gestational diabetes mellitus in twin pregnancies according to changes in its diagnostic criteria from National Diabetes Data Group criteria to Carpenter and Coustan criteria: a retrospective cohort study

**DOI:** 10.1186/s12884-021-04361-9

**Published:** 2022-01-03

**Authors:** Yejin Kim, Sir-yeon Hong, Seo-yeon Kim, Yoo-min Kim, Ji-Hee Sung, Suk-Joo Choi, Soo-young Oh, Cheong-Rae Roh

**Affiliations:** 1 Department of Obstetrics and Gynecology, Samsung Medical Center, Sungkyunkwan University School of Medicine, 81 Irwon-ro Gangnam-gu, Seoul, 06351 Korea; 2 Department of Obstetrics and Gynecology, Chung-Ang University College of Medicine, Seoul, Korea

**Keywords:** Pregnancy, twin, Diabetes, gestational, Pregnancy outcome

## Abstract

**Background:**

To compare obstetric and neonatal outcomes in twin pregnancies with or without gestational diabetes mellitus (GDM) before and after changes in GDM diagnostic criteria.

**Methods:**

This was a retrospective cohort study of 1,764 twin pregnancies including 130 women with GDM (GDM group) and 1,634 women without GDM (non-GDM group). Patients with pregestational diabetes, unknown GDM status, and fetal death at < 24 gestational weeks were excluded. Obstetric and neonatal outcomes were compared between the two groups by two periods: period 1 (1995–2005) and period 2 (2005–2018) when National Diabetes Data Group criteria and Carpenter and Coustan criteria were used for diagnosis of GDM, respectively.

**Results:**

The incidence of GDM in twin pregnancies increased from 4.0% in period 1 to 9.3% in period 2. Composite obstetric complications rate was significantly higher in the GDM group than that in the non-GDM group during period 1 (72.0% vs. 45.5%, *P* = 0.009). However, it became comparable during period 2 (60.0% vs. 57.4%, *P* = 0.601). Interaction between GDM and period indicated a significant differential effect of GDM by period on the rate of composite obstetric complications. The rate of composite neonatal complications was similar between the two groups during both periods. The interaction between GDM and period was not significant.

**Conclusion:**

After changes of GDM diagnostic criteria, the incidence of GDM increased more than twice, and the rate of composite obstetric complications decreased, but the rate of composite neonatal complications did not change significantly.

## Background

Twin pregnancy is increasing worldwide with a shift toward an older maternal age and an increasing use of assisted reproductive technology (ART) [1–3]. The incidence of twin pregnancy in Korea increased from 2.7% in 2008 to 4.1% by 2018 [4]. Obstetric and perinatal complications are more common in women with twin pregnancy, including hyperemesis, miscarriage, gestational diabetes mellitus (GDM), hypertension, anemia, placenta previa, placenta abruptio, preterm labor, preterm premature rupture of membranes (PPROM), preterm delivery (PTD), cesarean section, and fetal and infant morbidity and mortality [2, 5, 6].

GDM is defined as a glucose tolerance disorder that is first diagnosed during pregnancy [7]. Women with GDM have higher risks of preeclampsia, polyhydramnios, PPROM, preterm labor, PTD, cesarean section, and development of diabetes later in life [7]. Furthermore, GDM is associated with a higher risk of macrosomia, neonatal hypoglycemia, hyperbilirubinemia, shoulder dystocia, and birth trauma [7]. The incidence of GDM is increasing worldwide [8, 9]. In Korea, the incidence of GDM increased from 5.7% in 2009 to 14.9% by 2016 [10, 11]. This increase is associated with a greater rate of obesity in women of reproductive age, older maternal age, and a trend of lowering diagnostic criteria thresholds for GDM [12, 13].

Screening and diagnosis of GDM have been evolved through several decades [7, 12]. However, guidelines for screening and diagnosing GDM still vary among countries and individual institutes. They also vary among major societies worldwide [14]. Currently, two alternate methods are commonly used for screening and diagnosing GDM in Korea: 1) a one-step approach of a diagnostic 2-h 75-g oral glucose tolerance test (OGTT); and 2) a two-step approach of a 1-h 50-g OGTT followed by a diagnostic 4-h 100-g OGTT. There are two main different threshold levels with the 100-g OGTT: the Carpenter and Coustan (C–C) criteria and the National Diabetes Data Group (NDDG) criteria. The C–C criteria have lower cut-off levels than the NDDG criteria. The incidence of GDM increases about 1.5 times when using the C–C criteria compared with the NDDG criteria [7, 15]. In our center, we diagnosed GDM only using the two-step method, and the threshold levels was changed from the NDDG criteria to C–C criteria.

Concurrent presentation of GDM and twin pregnancy is increasing with growing prevalence of GDM and twin pregnancy [5]. However, current guidelines for diagnosis and treatment of GDM are mainly based on data of singleton pregnancies. Although the diagnosis, treatment, and prognosis of GDM in twin pregnancies might be different from those in singleton pregnancies, most of previous studies have focused on GDM in singleton pregnancies. Moreover, there are limited data regarding outcomes of GDM in twin pregnancies according to different diagnostic criteria. Therefore, the main aim of this study was to investigate effects of changes in GDM diagnostic criteria on obstetric and neonatal outcomes in twin pregnancies. Obstetric and neonatal outcomes in twin pregnancies according to GDM status in each period were also investigated.

## Materials and Methods

This was a retrospective cohort study including all twin pregnant women who delivered at ≥ 24 weeks of gestation between January 1995 and December 2018 in Samsung Medical Center, Seoul, South Korea. Patients with pregestational diabetes, unknown GDM status such as preterm delivery before GDM screening test, and fetal death in utero (FDIU) < 24 gestational weeks were excluded. This study was approved by the Institutional Review Board for Clinical Research (No. 2019–07-159).

Screening and diagnosis of GDM were done with a two-step approach: 50-g OGTT followed by 100-g OGTT between 24 and 28 weeks of gestation. During the study period, there was a main change in the screening and diagnosis of GDM. Before December 2005 (period 1), women with 50-g OGTT level ≥ 140 mg/dl (7.8 mmol/L) underwent 100-g OGTT with GDM diagnosed when the following two or more of plasma glucose levels were above the NDDG criteria: fasting ≥ 105 mg/dl (5.8 mmol/L), 1-h ≥ 190 mg/dl (10.6 mmol/L), 2-h ≥ 165 mg/dl (9.2 mmol/L), and 3-h ≥ 145 mg/dl (8.0 mmol/L). After December 2005 (period 2), the cut-off criteria (≥ 140 mg/dl (7.8 mmol/L) in low-risk women and ≥ 130 mg/dl (7.2 mmol/L) (in high-risk women) of the 50-g OGTT were used. High-risk women were those with obesity, history of GDM or macrosomia during previous pregnancy, family history of type II DM, obesity, and repeated glycosuria. Women who had a 50-g OGTT level above the cut-off underwent the 100-g OGTT with GDM diagnosed when the following two or more of plasma glucose levels were above the C–C criteria: fasting ≥ 95 mg/dl (5.3 mmol/L), 1-h ≥ 180 mg/dl (10.0 mmol/L), 2-h ≥ 155 mg/dl (8.6 mmol/L), and 3-h ≥ 140 mg/dl (7.8 mmol/L).

Women who were diagnosed as GDM in our hospital were managed with nonpharmacologic approaches of dietary modifications and exercise. Women with GDM were encouraged to do daily self-monitoring of blood glucose (SMBG) by checking fasting blood glucose (FBS) level and 1-h postprandial (PP1) glucose level. Pharmacologic treatment using metformin or insulin treatment was recommended when FBS levels were consistently greater than or equal to 95 mg/dL (5.3 mmol/L) or PP1 glucose levels were consistently greater than or equal to 140 mg/dL (7.8 mmol/L).

Maternal characteristics and obstetric and neonatal outcomes were obtained by reviewing their medical records. Maternal characteristics included maternal age, parity, pre-pregnancy body weight, height, and BMI, weight gain during pregnancy, history of previous PTD, ART conception, and chorionicity. Chorionicity was evaluated by prenatal ultrasound and confirmed after delivery by obstetricians and by pathology reports, where available. Obstetric outcomes included twin-to-twin transfusion syndrome (TTTS), preterm labor, incompetent internal os of cervix (IIOC), PPROM, preeclampsia, placenta previa, placenta abruption, fetal congenital anomaly, FDIU ≥ 24 weeks of gestation, gestational age at delivery, *PTD* < 34 weeks and *PTD* < 37 weeks of gestation, cesarean section, and birth weight discordancy of 20% or more. Composite obstetric complications were defined as having one or more of preterm labor, PPROM, preeclampsia, *PTD* < 34 weeks of gestation, FDIU ≥ 24 weeks of gestation, and fetal congenital anomaly.

Neonatal outcomes included birth weight, sex, 1-min and 5-min Apgar scores, neonatal intensive care unit (NICU) admission, mechanical ventilation, respiratory distress syndrome (RDS), bronchopulmonary dysplasia (BPD), neonatal sepsis, transient tachypnea of the newborn (TTN), hypoglycemia, hyperbilirubinemia, and neonatal mortality. Large for gestational age (LGA) and small for gestational age (SGA) were defined as neonatal birth weight > 90th and < 10th centiles for gestational age, respectively, based on birth weight standards adjusted for gestational age and plurality from a Korean national database [16]. Composite neonatal complications were defined as having one or more of NICU admission, 1-min Apgar score < 4, 5-min Apgar score < 7, LGA, RDS, BPD, TTN, sepsis, hypoglycemia, hyperbilirubinemia, and neonatal death.

Maternal characteristics and obstetric outcomes between the GDM group and the non-GDM group were compared using two-sample Student’s *t*-test for continuous variable and Chi-square test or Fisher's exact test for categorical variables, as appropriate. Neonatal outcomes of twin pairs between the GDM group and the non-GDM group were compared using generalized estimating equations (GEE). Interaction tests between GDM and period were performed to assess possible differential effect of GDM on maternal and neonatal outcomes according to changes in the diagnostic criteria of GDM: logistic model (categorical variables) or regression model (continuous variables) was used to analyze maternal characteristics and obstetric outcomes and GEE was used for neonatal outcomes. A two-tailed *P*-value below 0.05 was considered statistically significant. All statistical analyses were carried out using the Statistical Package for Social Sciences version 25 (SPSS Statistics; IBM, Armonk, NY, USA) and SAS version 9.4 (SAS Institute, Cary, NC, USA).

## Results

**Fig. 1:**
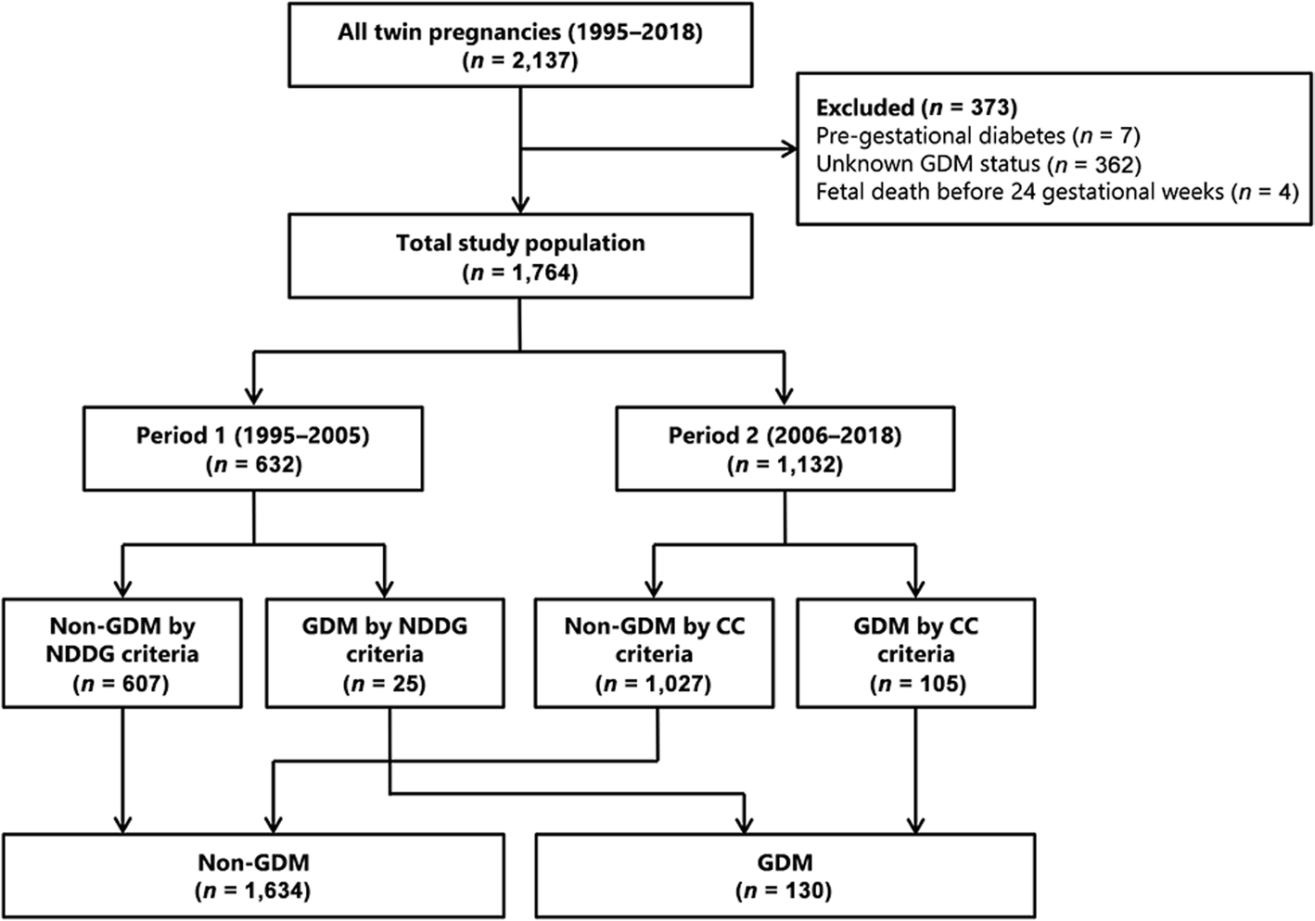
Overview of patient selection and classification of the study population

During the study period, 2,137 women with twin pregnancies delivered at ≥ 24 weeks of gestation in our institute. After excluding 373 women based on the exclusion criteria, a total of 1,764 twin pregnant women were included in the analysis (Fig. 1). The incidence of GDM in twin pregnancies in our cohort was 7.4% (130/1,764). Among them, 25/632 (4.0%) women were diagnosed as GDM by the NDDG criteria during period 1 and 105/1,132 (9.3%) women were diagnosed as GDM by the C–C criteria during period 2 (Fig. 1).

Results of comparisons of maternal characteristics between the non-GDM group and the GDM group during each period are summarized in Table 1. Maternal age was significantly higher in the GDM group than in the non-GDM group during both periods 1 and 2. Pre-pregnancy BMI was significantly higher in the GDM group than in the non-GDM group during period 2 only, whereas gestational weight gain was significantly lower in the GDM group than in the non-GDM group during both periods 1 and 2. The rate of history of PTD was significantly higher in the GDM group than in the non-GDM group during period 1 only. The rate of dichorionic twins was significantly higher in the GDM group than in the non-GDM group during period 2 only. However, the interaction between GDM and period was not significant for any outcome.

**Table 1:** Maternal characteristics of subjects in non-GDM and GDM groups before (period 1) and after (period 2) changes of GDM diagnostic criteria

	**Period 1 (NDDG criteria)**			**Period 2 (C–C criteria)**			**Interaction test** *P*-value^*^
	**Non-GDM** **(*n* = 607)**	**GDM** **(*n* = 25)**	***P*-value**	**Non-GDM** **(*n* = 1,027)**	**GDM** **(*n* = 105)**	***P*-value**	
Maternal age (year)	31.1 ± 3.8	33.9 ± 4.2	0.001	32.8 ± 3.6	34.1 ± 3.7	< 0.001	0.068
Maternal age ≥ 35 years	102 (16.8)	11 (44.0)	0.002	321 (31.3)	48 (45.7)	0.003	0.111
Pre-pregnancy BMI (Kg/m^2^)	20.6 ± 2.7	22.6 ± 4.5	0.119	21.0 ± 3.0	22.3 ± 3.8	0.001	0.368
Gestational weight gain (Kg)	17.6 ± 5.8	12.7 ± 4.9	< 0.001	15.7 ± 6.2	12.3 ± 5.1	< 0.001	0.293
Multiparity	143 (23.6)	9 (36.0)	0.154	231 (22.5)	25 (23.8)	0.759	0.281
History of PTD	19 (3.1)	4 (16.0)	0.010	35 (3.4)	6 (5.7)	0.264	0.099
ART pregnancy	268 (44.2)	11 (44.0)	0.988	495 (48.3)	61 (58.1)	0.055	0.381
Chorionicity			0.705			0.024	0.722
Monoamnionic	5 (0.8)	0 (0)		2 (0.2)	1 (1.0)		
Monochorionic diamnionic	163 (26.9)	5 (20.0)		198 (19.3)	11 (10.5)		
Dichorionic diamnionic	430 (70.8)	19 (76.0)		827 (80.5)	93 (88.6)		
Unknown	9 (1.5)	1 (4.0)		0 (0)	0 (0)		
Insulin treatment	0 (0)	5 (20.0)	NA	0 (0)	24 (23.1)	NA	NA

Data expressed as mean ± standard deviation or number (%). *NDDG* National Diabetes Data Group, *C–C* Carpenter and Coustan, *GDM* gestational diabetes mellitus, *PTD* preterm delivery, *ART* assisted reproductive technology, *NA* not analyzable. ^*^logistic model for categorical variables and regression model for continuous variables

The rate of PPROM was significantly higher in the GDM group than in the non-GDM group during period 2, but not during period 1 (Table 2). However, the interaction between GDM and period was not significant. Mean gestational age at delivery was significantly lower while rates of *PTD* < 34 weeks and *PTD* < 37 weeks of gestation were significantly higher in the GDM group than in the non-GDM group during period 1, but not during period 2. Interaction between GDM and period indicated a significant differential effect of GDM by period on the rate of *PTD* < 34 weeks of gestation. Cesarean section rates were comparable between GDM and non-GDM groups during both periods 1 and 2. However, the interaction between GDM and period showed that the effect of GDM on cesarean section rate varied across periods. The rate of composite obstetric complications was significantly higher in the GDM group than in the non-GDM group during period 1, but was comparable during period 2. The interaction between GDM and period indicated a significant different effect of GDM by period on the rate of composite obstetric complications.

**Table 2:** Obstetric outcomes of non-GDM and GDM groups before (period 1) and after (period 2) changes of GDM diagnostic criteria

	**Period 1 (NDDG criteria)**			**Period 2 (C–C criteria)**			**Interaction test** *P*-value^*^
	**Non-GDM** **(*n* = 607)**	**GDM** **(*n* = 25)**	***P*-value**	**Non-GDM** **(*n* = 1,027)**	**GDM** **(*n* = 105)**	***P*-value**	
TTTS	6 (1.0)	0 (0)	> 0.999	15 (1.5)	0 (0)	0.386	0.999
Preterm labor	163 (26.9)	9 (36.0)	0.314	341 (33.2)	34 (32.4)	0.865	0.333
IIOC	3 (0.5)	0 (0)	> 0.999	33 (3.2)	3 (2.9)	> 0.999	0.977
PPROM	87 (14.3)	7 (28.0)	0.079	182 (17.7)	33 (31.4)	0.001	0.863
Preeclampsia	55 (9.1)	1 (4.0)	0.716	120 (11.7)	12 (11.4)	0.938	0.433
Placenta previa	11 (1.8)	0 (0)	> 0.999	27 (2.6)	1 (1.0)	0.507	0.976
Placenta abruptio	12 (2.0)	0 (0)	> 0.999	27 (2.6)	5 (4.8)	0.210	0.971
Fetal congenital anomaly^a^	16 (2.6)	1 (4.0)	0.501	87 (8.5)	6 (5.7)	0.327	0.453
FDIU^a^	12 (2.0)	1 (4.0)	0.411	14 (1.4)	1 (1.0)	> 0.999	0.464
GA at delivery (weeks)	35.4 ± 2.5	34.2 ± 3.0	0.028	35.2 ± 3.2	35.3 ± 2.7	0.461	0.066
PTD < 34 weeks	118 (19.4)	10 (40.0)	0.012	247 (24.1)	25 (23.8)	0.956	0.034
PTD < 37 weeks	451 (74.3)	23 (92.0)	0.045	563 (54.8)	63 (60.0)	0.309	0.130
Cesarean section	568 (93.6)	21 (84.0)	0.082	888 (86.5)	94 (89.5)	0.379	0.047
Birth weight discordancy ≥ 20%	109 (18.0)	6 (24.0)	0.431	218 (21.2)	18 (17.1)	0.327	0.254
Composite obstetric complications^b^	276 (45.5)	18 (72.0)	0.009	589 (57.4)	63 (60.0)	0.601	0.042

Data expressed as mean ± standard deviation or number (%). *NDDG* National Diabetes Data Group, *C–C* Carpenter and Coustan, *GDM* gestational diabetes mellitus, *TTTS* twin-twin transfusion syndrome, *IIOC* incompetent internal os of cervix, *PPROM* preterm premature rupture of membranes, *FDIU* fetal death in utero, *GA* gestational age, *PTD* preterm delivery. ^*^logistic model for categorical variables and regression model for continuous variables. ^a^one or more twin in twin pairs. ^b^defined as having one or more of preterm labor, *PPROM*, preeclampsia, *PTD* < 34 weeks, *FDIU*, and fetal congenital anomaly

Table 3 shows results of comparisons of neonatal outcomes between the non-GDM group and the GDM group during each period. Rates of RDS were comparable between GDM and non-GDM groups during both periods 1 and 2. However, trends of the rate of RDS for periods 1 and 2 according to GDM status were opposite to each other. This difference was statistically significant in the interaction test. The rate of hypoglycemia was significantly higher while the rate of hyperbilirubinemia was significantly lower in the GDM group than in the non-GDM group during period 2, but not during period 1. However, the interaction between GDM and period was significant for the rate of hyperbilirubinemia only. Rates of composite neonatal complications were not significantly different between GDM and non-GDM groups during period 1 or 2. The interaction between GDM and period was not significant either.

**Table 3:** Neonatal outcome of non-GDM and GDM groups before (period 1) and after (period 2) changes of GDM diagnostic criteria

	**Period 1 (NDDG criteria)**			**Period 2 (C–C criteria)**			**Interaction test** *P*-value^*^
	**Non-GDM** **(*n* = 1,213)**	**GDM** **(*n* = 50)**	***P*-value**	**Non-GDM** **(*n* = 2,053)**	**GDM** **(*n* = 210)**	***P*-value**	
Sex (male)	605 (49.9)	30 (60.0)	0.198	1,054 (51.3)	108 (51.4)	0.981	0.253
Birth weight (Kg)	2.2 ± 0.5	2.1 ± 0.6	0.120	2.2 ± 0.6	2.3 ± 0.5	0.570	0.100
SGA	95 (7.8)	3 (6.0)	0.626	195 (9.5)	14 (6.7)	0.224	0.882
LGA	130 (10.7)	8 (16.0)	0.201	220 (10.7)	17 (8.1)	0.251	0.087
1-min Apgar score < 4	48 (4.0)	5 (10.2)	0.080	31 (1.5)	7 (3.4)	0.084	0.797
5-min Apgar score < 7	51 (4.2)	3 (6.1)	0.633	38 (1.9)	5 (2.4)	0.589	0.888
NICU admission	518 (43.1)	24 (49.0)	0.605	735 (36.0)	80 (38.5)	0.627	0.807
Mechanical ventilator	174 (14.5)	11 (22.5)	0.262	369 (18.1)	29 (13.9)	0.228	0.114
RDS	121 (10.1)	10 (20.4)	0.077	275 (13.5)	20 (9.6)	0.229	0.033
BPD	29 (2.4)	0 (0%)	NA	100 (4.9)	7 (3.4)	0.414	NA
Sepsis	83 (6.9)	5 (10.2)	0.370	129 (6.3)	11 (5.3)	0.576	0.299
TTN	43 (3.6)	2 (4.1)	0.856	26 (1.3)	2 (1.0)	0.699	0.686
Hypoglycemia	72 (6.0)	2 (4.1)	0.565	36 (1.8)	10 (4.8)	0.020	0.088
Hyperbilirubinemia	330 (27.5)	19 (38.8)	0.194	543 (26.6)	37 (17.8)	0.027	0.023
Neonatal death	16 (1.3)	0 (0)	NA	26 (1.3)	3 (1.4)	0.840	NA
Composite neonatal complications^a^	638 (53.1)	29 (59.2)	0.533	1,059 (51.2)	105 (50.5)	0.726	0.473

Data expressed as mean ± standard deviation or number (%). *NDDG* National Diabetes Data Group, *C–C* Carpenter and Coustan, *GDM* gestational diabetes mellitus, *SGA* small for gestational age, *LGA* large for gestational age, *NICU* neonatal intensive care unit, *RDS* respiratory distress syndrome, *BPD* bronchopulmonary dysplasia, *TTN* transient tachypnea of the newborn, *NA* not analyzable. ^*^generalized estimating equations. ^a^defined as having one or more of NICU admission, 1-min AS (Apgar score) < 4, 5-min AS < 7, LGA, RDS, BPD, TTN, sepsis, hypoglycemia, hyperbilirubinemia, and death

## Discussion

This study compared obstetric and neonatal outcomes in twin pregnancies with or without GDM before and after changes in the diagnostic criteria of GDM from NDDG criteria to C–C criteria. We found that the incidence of GDM increased more than twice after changes of diagnostic criteria. The rate of composite obstetric complications was significantly higher in the GDM group than in the non-GDM group when NDDG criteria was used, but it became comparable between the two groups after changing to C–C criteria. However, the rate of composite neonatal complications did not change significantly before and after the change of diagnostic criteria.

The risk of developing GDM during pregnancy might be variable according to race, age, nutrition, pre-pregnancy weight or BMI, familial history, and hormonal and genetic factors [8, 9, 12, 13, 17, 18]. There are conflicting data regarding whether women with twin pregnancies have a higher risk of GDM than women with singleton pregnancies. Some studies have suggested that the risk of GDM increases with twin pregnancy [6, 19, 20], while other studies have shown no significant differences in GDM incidence according to the number of fetus [21, 22]. Maternal age is also associated with an increased risk of GDM [23, 24]. In the present study, maternal age was significantly higher in the GDM group than that in the non-GDM group. Higher pre-pregnancy BMI is a well-known risk factor of GDM [25]. In the present study, pre-pregnancy BMI was higher in the GDM group than in the non-GDM group. Furthermore, gestational weight gain was significantly lower in the GDM group in our study. This may be due to intentional life style changes after the diagnosis of GDM.

The most important factor associated with an increase in the incidence of GDM was the use of lower diagnostic cut-off criteria in our study. Previous studies have shown that the incidence of GDM is increased about 1.5 times when using the C–C criteria compared to that using the NDDG criteria [15]. The incidence of GDM increased about 2 to 3 times when using the one-step approach of 75 g 2-h OGTT with International Association of the Diabetes and Pregnancy Study Groups (IADPSG) criteria compared to that when using the two-step approach with the NDDG criteria [26, 27]. However, these studies were based on data of singleton pregnancies. There were limited data for twin pregnancies. A retrospective cohort study of 1,461 twin pregnancies showed that the incidence of GDM increased threefold after changing the method of screening and diagnosis of GDM from the standard two-step approach (50 g screening test followed by a 100 g diagnostic OGTT utilizing C–C criteria) to the IADPSG protocol [28]. In our center, we used the two-step approach for diagnosis of GDM. The incidence of GDM increased from 4.0% to 9.3% when the diagnostic criteria of GDM with 100-g GTT were changed from the NDDG criteria to the C–C criteria. To the best of our knowledge, the current study was the first to evaluate the change in incidence of GDM according to changes in diagnostic criteria of GDM from NDDG criteria to C–C criteria in twin pregnancies.

The purpose of using lower diagnostic cut-off criteria so that more women could be diagnosed with GDM is to reduce obstetric and neonatal complications associated with GDM. It is well-known that hyperglycemia is associated with a higher risk of adverse pregnancy outcomes in singleton pregnancies [7]. However, previous studies have shown conflicting results in twin pregnancies. Some previous studies have reported that GDM does not increase the risk of adverse pregnancy outcomes in twin pregnancies [5, 29, 30], whereas other studies have shown that GDM is associated with an increased risk of maternal adverse outcome and neonatal adverse outcome [6, 31, 32]. In the present study, GDM in twin pregnancy was associated with a higher rate of composite obstetric complications in the GDM group than in the non-GDM group. However, the rate of composite obstetric complications was only significantly higher during period 1 when NDDG criteria were used. It became comparable between the GDM group and the non-GDM group during period 2 when NDDG criteria were used.

There are limited data on changes of obstetric and neonatal outcomes according to changes in the diagnostic criteria of GDM from the NDDG criteria to the C–C criteria. Some studies have compared pregnancy outcomes between untreated GDM diagnosed by the C–C criteria and treated GDM diagnosed by the NDDG criteria [33–35]. These studies showed that untreated GDM diagnosed by C–C criteria was associated with an increased risk of preeclampsia and similar or slight increased risks of cesarean delivery and macrosomia [33, 34]. Another study showed that not only a severe GDM group diagnosed by the NDDG criteria, but also a milder GDM group diagnosed by only the C–C criteria benefited from active intervention such as nutritional counseling and insulin therapy when indicated in singleton pregnancies [35]. However, there are insufficient data on the effect of changes in the diagnostic criteria of GDM on pregnancy outcome in twin pregnancies. Only one retrospective study of 1461 twin pregnancies showed that using IADPSG screening method resulted in a 38% lower risk of pre-eclampsia compared with using a standard two-step approach (50 g screening test followed by a 100 g diagnostic OGTT utilizing the C–C criteria) [28].

A higher rate of composite obstetric complications in the GDM group in our study was mainly due to higher rates of PPROM and PTD. The rate of PPROM was higher in the GDM group than in the non-GDM group during both periods, although it was not significantly higher during period 1. Recent studies have shown a significant association between GDM and PPROM [36, 37]. Although the exact reason for the higher risk of PPROM in women with GDM is not fully understood yet, inflammation might be responsible for the link between GDM and PPROM [38]. A higher rate of PTD history and older maternal age in the GDM group might be associated with the increased risk of PPROM in our study. However, the interaction between GDM and period indicated no significant differential effect of GDM by period on the incidence of PPROM in our study. Nevertheless, gestational age at delivery and rates of *PTD* < 34 weeks and *PTD* < 37 weeks of gestation improved after changes in the diagnostic criteria of GDM, although a significant differential effect of GDM by period was found only in the rate of *PTD* < 34 weeks of gestation.

In our study, GDM in twin pregnancy was not associated with an increased risk of neonatal adverse outcome during period 1. Neonatal hypoglycemia was significantly higher in the GDM group during period 2, consistent with other previous studies [39, 40]. However, there was no significant differential effect of GDM by period. Interestingly, neonatal hyperbilirubinemia became significantly lower in the GDM group during period 2 and the rate of RDS was significantly decreased after changes of diagnostic criteria of GDM from NDDG criteria to C–C criteria. These findings were consistent with other previous studies showing that more diagnosis and treatment using lower diagnostic cut-off criteria improved the overall neonatal outcome [26, 41]. However, the rate of composite neonatal complications was not significantly different between the GDM group and the non-GDM group during either period. The interaction between GDM and period was not significant either. Further studies are needed to clarify the exact association between improved neonatal outcomes and changes of diagnostic criteria of GDM.

The strength of our study was that it had a large sample size of 1,764 twin pregnant women during a 24-year study period. However, our sample size was still insufficient to have an adequate power because numbers of twin pregnant women with GDM were too low, especially during period 1 (*n* = 25). In addition, because our data were accumulated for 24 years, there might be significant changes in management protocols about twin pregnancy and GDM that were not adequately controlled in our analyses. For example, at our institution, elective cesarean delivery of twin pregnancies was commonly performed at 34–36 weeks of gestation before the mid-2000s. However, after the mid-2000s, since late preterm birth and optimal gestational age of delivery for twin pregnancies have drawn increasing attention, late-preterm elective cesarean twin deliveries have decreased [42]. This is the main reason for the decreased rate of *PTD* < 37 weeks of gestation in our study. Nevertheless, a significant trend of decreased rate of *PTD* < 34 weeks of gestation after changes in the diagnostic criteria of GDM might still be significant because the goal of this protocol was to reduce PTD at 34–36 weeks of gestation. This protocol change was not differently adopted for women with or without GDM.

This study is further limited by the inherent nature of a retrospective study design that limited our ability to control other unknown potential confounding factors. In our study population, maternal characteristics, such as maternal age, pre-pregnancy BMI, gestational weight gain, history of PTD, and chorionicity, were not comparable between the non-GDM group and the GDM group during each period. However, the interaction test did not show a significant different effect of GDM by period on any of maternal characteristics. In addition, although this study included a large sample size, it contained only patients from a single tertiary hospital. These subjects cannot represent the total Korean population.

## Conclusion

The incidence of GDM increased more than twice after changes in the diagnostic criteria of GDM from the NDDG criteria to the C–C criteria in our study population. The changes in the diagnostic criteria of GDM might be associated with a reduction in the rate of composite obstetric complications, especially in the rate of *PTD* < 34 weeks of gestation. Although there was no significant change in the rate of composite neonatal complications between the two periods, rates of neonatal hyperbilirubinemia and RDS decreased after changes in the diagnostic criteria of GDM. However, more studies with larger sample size are needed to better understand the role of strict diagnostic criteria for twin pregnancy with GDM and its effect on pregnancy outcomes.

## Data Availability

The datasets used and/or analysed during the current study are available from the corresponding author on reasonable request.

## Abbreviations

GDM: Gestational diabetes mellitus
ART: Assisted reproductive technology
PPROM: Preterm premature rupture of membranes
PTD: Preterm delivery
The C–C criteria: The Carpenter and Coustan criteria
The NDDG criteria: The National Diabetes Data Group criteria
FDIU: Fetal death in utero
SMBG: Self-monitoring of blood glucose
FBS: Fasting blood glucose
PP1: 1-Hour postprandial
TTTS: Twin-to-twin transfusion syndrome
IIOC: Incompetent internal os of cervix
NICU: Neonatal intensive care unit
RDS: Respiratory distress syndrome
BPD: Bronchopulmonary dysplasia
TTN: Transient tachypnea of the newborn
LGA: Large for gestational age
SGA: Small for gestational age
GEE: Generalized estimating equations
IADPSG: International Association of the Diabetes and Pregnancy Study Groups

## References

[1] Blondel B, Kaminski M (2002). Trends in the occurrence, determinants, and consequences of multiple births. Semin Perinatol.

[2] Committee on Practice Bulletins-Obstetrics, Society for Maternal-Fetal Medicine (2016). Practice Bulletin No. 169: Multifetal Gestations: Twin, Triplet, and Higher-Order Multifetal Pregnancies. Obstet Gynecol.

[3] Park YS, Choi SH, Shim KS, Chang JY, Hahn WH, Choi YS (2010). Multiple births conceived by assisted reproductive technology in Korea. Korean J Pediatr.

[4] Korean Statistical Information Service (2019). Birth data for 2018, Korea.

[5] McGrath RT, Hocking SL, Scott ES, Seeho SK, Fulcher GR, Glastras SJ (2017). Outcomes of twin pregnancies complicated by gestational diabetes: a meta-analysis of observational studies. J Perinatol.

[6] Rauh-Hain JA, Rana S, Tamez H, Wang A, Cohen B, Cohen A (2009). Risk for developing gestational diabetes in women with twin pregnancies. J Matern Fetal Neonatal Med.

[7] Committee on Practice Bulletins-Obstetrics (2018). ACOG Practice Bulletin No. 190: Gestational Diabetes Mellitus. Obstet Gynecol.

[8] Ferrara A (2007). Increasing prevalence of gestational diabetes mellitus: a public health perspective. Diabetes Care.

[9] Anna V, van der Ploeg HP, Cheung NW, Huxley RR, Bauman AE (2008). Sociodemographic correlates of the increasing trend in prevalence of gestational diabetes mellitus in a large population of women between 1995 and 2005. Diabetes Care.

[10] Koo BK, Lee JH, Kim J, Jang EJ, Lee CH (2016). Prevalence of Gestational Diabetes Mellitus in Korea: A National Health Insurance Database Study. PLoS One.

[11] Jung CH, Jung SH, Choi D, Kim BY, Kim CH, Mok JO (2021). Gestational diabetes in Korea: Temporal trends in prevalence, treatment, and short-term consequences from a national health insurance claims database between 2012 and 2016. Diabetes Res Clin Pract.

[12] Moses RG, Morris GJ, Petocz P, San Gil F, Garg D (2011). The impact of potential new diagnostic criteria on the prevalence of gestational diabetes mellitus in Australia. Med J Aust.

[13] Vahratian A (2009). Prevalence of overweight and obesity among women of childbearing age: results from the 2002 National Survey of Family Growth. Matern Child Health J.

[14] Bhavadharini B, Uma R, Saravanan P, Mohan V (2016). Screening and diagnosis of gestational diabetes mellitus - relevance to low and middle income countries. Clin Diabetes Endocrinol.

[15] Ferrara A, Hedderson MM, Quesenberry CP, Selby JV (2002). Prevalence of gestational diabetes mellitus detected by the national diabetes data group or the carpenter and coustan plasma glucose thresholds. Diabetes Care.

[16] Hong JS, Yi SW, Han YJ, Park YW, Nam CM, Kang HC (2007). Fetal growth and neonatal mortality in Korea. Paediatr Perinat Epidemiol.

[17] Galtier F (2010). Definition, epidemiology, risk factors. Diabetes Metab.

[18] Wong VW (2012). Gestational diabetes mellitus in five ethnic groups: a comparison of their clinical characteristics. Diabet Med.

[19] Lai FY, Johnson JA, Dover D, Kaul P (2016). Outcomes of singleton and twin pregnancies complicated by pre-existing diabetes and gestational diabetes: A population-based study in Alberta, Canada, 2005–11. J Diabetes.

[20] Retnakaran R, Shah BR (2016). Impact of Twin Gestation and Fetal Sex on Maternal Risk of Diabetes During and After Pregnancy. Diabetes Care.

[21] Yogev Y, Eisner M, Hiersch L, Hod M, Wiznitzer A, Melamed N (2014). The performance of the screening test for gestational diabetes in twin versus singleton pregnancies. J Matern Fetal Neonatal Med.

[22] Buhling KJ, Henrich W, Starr E, Lubke M, Bertram S, Siebert G (2003). Risk for gestational diabetes and hypertension for women with twin pregnancy compared to singleton pregnancy. Arch Gynecol Obstet.

[23] Pinheiro RL, Areia AL, Mota Pinto A, Donato H (2019). Advanced Maternal Age: Adverse Outcomes of Pregnancy. A Meta-Analysis Acta Med Port.

[24] Lee YJ, Kim MN, Kim YM, Sung JH, Choi SJ, Oh SY (2019). Perinatal outcome of twin pregnancies according to maternal age. Obstet Gynecol Sci.

[25] Garrison A (2015). Screening, diagnosis, and management of gestational diabetes mellitus. Am Fam Physician.

[26] Metzger BE, Lowe LP, Dyer AR, Trimble ER, Chaovarindr U, Group HSCR (2008). Hyperglycemia and adverse pregnancy outcomes. N Engl J Med.

[27] Waters TP, Dyer AR, Scholtens DM, Dooley SL, Herer E, Lowe LP (2016). Maternal and Neonatal Morbidity for Women Who Would Be Added to the Diagnosis of GDM Using IADPSG Criteria: A Secondary Analysis of the Hyperglycemia and Adverse Pregnancy Outcome Study. Diabetes Care.

[28] Liu X, Chen Y, Zhou Q, Shi H, Cheng WW (2015). Utilization of International Association of Diabetes and Pregnancy Study Groups criteria vs. a two-step approach to screening for gestational diabetes mellitus in Chinese women with twin pregnancies. Diabet Med.

[29] Okby R, Weintraub AY, Sergienko R, Eyal S (2014). Gestational diabetes mellitus in twin pregnancies is not associated with adverse perinatal outcomes. Arch Gynecol Obstet.

[30] Luo ZC, Simonet F, Wei SQ, Xu H, Rey E, Fraser WD (2011). Diabetes in pregnancy may differentially affect neonatal outcomes for twins and singletons. Diabet Med.

[31] Simoes T, Queiros A, Correia L, Rocha T, Dias E, Blickstein I (2011). Gestational diabetes mellitus complicating twin pregnancies. J Perinat Med.

[32] Foeller ME, Zhao S, Szabo A, Cruz MO (2015). Neonatal outcomes in twin pregnancies complicated by gestational diabetes compared with non-diabetic twins. J Perinatol.

[33] Ricart W, Lopez J, Mozas J, Pericot A, Sancho MA, Gonzalez N (2005). Potential impact of American Diabetes Association (2000) criteria for diagnosis of gestational diabetes mellitus in Spain. Diabetologia.

[34] Berggren EK, Boggess KA, Stuebe AM, Jonsson FM (2011). National Diabetes Data Group vs Carpenter-Coustan criteria to diagnose gestational diabetes. Am J Obstet Gynecol.

[35] Harper LM, Mele L, Landon MB, Carpenter MW, Ramin SM, Reddy UM (2016). Carpenter-Coustan Compared With National Diabetes Data Group Criteria for Diagnosing Gestational Diabetes. Obstet Gynecol.

[36] Sae-Lin P, Wanitpongpan P (2019). Incidence and risk factors of preterm premature rupture of membranes in singleton pregnancies at Siriraj Hospital. J Obstet Gynaecol Res.

[37] Bouvier D, Forest JC, Blanchon L, Bujold E, Pereira B, Bernard N (2019). Risk Factors and Outcomes of Preterm Premature Rupture of Membranes in a Cohort of 6968 Pregnant Women Prospectively Recruited. J Clin Med.

[38] Li S, Yang H (2019). Relationship between advanced glycation end products and gestational diabetes mellitus. J Matern Fetal Neonatal Med.

[39] Dinham GK, Henry A, Lowe SA, Nassar N, Lui K, Spear V (2016). Twin pregnancies complicated by gestational diabetes mellitus: a single centre cohort study. Diabet Med.

[40] Sheehan ACM, Umstad MP, Cole S, Cade TJ (2019). Does Gestational Diabetes Cause Additional Risk in Twin Pregnancy?. Twin Res Hum Genet.

[41] Crowther CA, Hiller JE, Moss JR, McPhee AJ, Jeffries WS, Robinson JS (2005). Effect of treatment of gestational diabetes mellitus on pregnancy outcomes. N Engl J Med.

[42] Hong JY, Lee HR, Kim Y, Kim YM, Sung JH, Choi SJ (2020). Changes in the perinatal outcomes of twin pregnancies delivered at a tertiary referral center in Korea during a 24-year period from 1995 to 2018. Obstet Gynecol Sci.

